# Circulating Ceramide: A New Cardiometabolic Biomarker in Patients With Comorbid Acute Coronary Syndrome and Type 2 Diabetes Mellitus

**DOI:** 10.3389/fphys.2020.01104

**Published:** 2020-09-15

**Authors:** Ruihua Cao, Zhiyi Fang, Sulei Li, Mengqi Xu, Jibin Zhang, Dong Han, Wenchao Hu, Liqiu Yan, Yabin Wang, Li Fan, Feng Cao

**Affiliations:** ^1^Department of Cardiology, The Second Medical Centre, Chinese PLA General Hospital, National Clinical Research Center for Geriatric Diseases, Beijing, China; ^2^The Second Medical Centre, Chinese PLA General Hospital, National Clinical Research Center for Geriatric Diseases, Beijing, China

**Keywords:** ceramide, acute coronary syndrome with type 2 diabetes mellitus, comorbidity, cardiometabolic diseases, risk factors

## Abstract

**Aims:**

This study investigated the association of circulating ceramides in patients with comorbid acute coronary syndrome and type 2 diabetes mellitus (ACS-DM).

**Methods:**

A total of 761 patients with coronary heart disease who were admitted to the Department of Cardiology at the Chinese PLA General Hospital from March to August 2018 were enrolled in this study. Of these 761 patients, 282 were diagnosed with acute coronary syndrome (ACS). We selected 65 patients with ACS-DM (ACS-DM group; mean age 64.88 years; 38 men) and 65 patients with ACS but without any comorbidities (ACS group; mean age 64.68 years; 38 men); the two groups were matched by age and sex. We determined four circulating ceramides in 130 plasma samples: Cer(d18:1/16:0), Cer(d18:1/18:0), Cer(d18:1/24:1), and Cer(d18:1/24:0). The ceramides in plasma samples from patients with ACS and those from patients with ACS-DM were compared. Pearson correlation coefficients between individual ceramides and traditional cardiovascular risk factors for the whole study population were calculated. Multiple logistic regression models were used to evaluate the relativity between the ceramide and ACS-DM.

**Results:**

Compared with the ACS group, the levels of Cer(d18:1/16:0), Cer(d18:1/18:0), and Cer(d18:1/24:1) and their ratios to Cer(d18:1/24:0) were higher in the ACS-DM group and Cer(d18:1/24:0) was lower in the ACS-DM group (*P* < 0.05). Correlation analysis demonstrated mild-to-moderate correlations of ceramide and traditional cardiovascular risk factors. There were relatively strong correlations of Cer(d18:1/18:0) and Cer(d18:1/24:1) with C-reactive protein, blood lipids, fasting blood glucose, and glycated hemoglobin A_1_c. In multiple logistic regression models, Cer(d18:1/18:0) [odds ratio (OR) 2.396; 95% confidence interval (CI) 1.103–5.205; *P* = 0.027], Cer(d18:1/24:1) (OR 2.826; 95% CI 1.158–6.896; *P* = 0.023), Cer(d18:1/18:0)/Cer(d18:1/24:0) (OR 2.242; 95% CI 1.103–4.555; *P* = 0.026), and Cer(d18:1/24:1)/Cer(d18:1/24:0) (OR 2.673; 95% CI 1.225–5.836; *P* = 0.014) were positively correlated with ACS-DM, and Cer(d18:1/24:0) (OR 0.200; 95% CI 0.051–0.778; *P* = 0.020) was negatively correlated with ACS-DM.

**Conclusion:**

Circulating ceramides are positively correlated with the risk of ACS-DM comorbidity. These results give a new insight into the pathogenesis of ACS-DM comorbidity and could provide new options for risk estimation.

## Introduction

The serum concentration of lipids is used to assess the risk of atherosclerotic heart disease. The level of low-density lipoprotein cholesterol (LDL-C) is closely related to atherosclerosis and acute coronary events. Previous studies have indicated that distinct ceramide species are closely related to cardiovascular death in patients with coronary heart disease (CHD). Ceramide molecules Cer(d18:1/16:0), which is abbreviated to Cer16:0; Cer(d18:1/18:0), which is abbreviated to Cer18:0; Cer(d18:1/24:1), which is abbreviated to Cer24:1; and their ratios to Cer(d18:1/24:0), which is abbreviated to Cer24:0, have been investigated as new risk stratification factors in patients with CHD (Meeusen et al., 2017; Peterson et al., 2018). In patients with CHD, a high level of Cer16:0, low level of Cer24:0, and a high Cer16:0/Cer24:0 ratio in plasma are related directly to cardiovascular mortality (Tarasov et al., 2014; Laaksonen et al., 2016). Accumulating evidence has shown that ceramides are closely related to the pathological process of type 2 diabetes mellitus (DM) and its complications (Galadari et al., 2013; Muilwijk et al., 2020).

Coronary heart disease is the main cause of mortality worldwide, resulting in more than 7 million deaths every year. Acute coronary syndrome (ACS) is a severe type of CHD associated with substantial mortality. DM is the main risk factor contributing to CHD, being regarded as having an equivalent risk to CHD (Juutilainen et al., 2005). Over 425 million people worldwide have DM. The number of adults in China with DM has reached 114 million, and China remains the country with the largest number of patients with DM (Cho et al., 2018). Cardiovascular events in patients with CHD and DM are five to six times higher than in patients with CHD alone (Høfsten et al., 2009).

Coronary heart disease and DM are both metabolic diseases and have mutual pathological mechanisms, but the pathological mechanism of the comorbidity is still unclear. Abnormal glucose and lipid metabolism is the pathological basis of many metabolic diseases. The pathophysiological alterations related to DM promote the development of CHD, the important driving mechanisms of which include oxidative stress, alterations in calcium metabolism, the inflammatory response, endothelial dysfunction, and autophagy (Yahagi et al., 2016; Zhang et al., 2018). Although our understanding of the pathogenesis of this comorbidity is increasing, the incidence of cardiovascular death in patients with acute coronary syndrome and type 2 diabetes mellitus (ACS-DM) remains high, and there is a lack of more effective risk stratification and prediction biomarkers for patients with ACS-DM.

It is not known whether the levels of ceramides are higher in patients with comorbid ACS-DM. Therefore, we measured the plasma concentrations of ceramides in patients with ACS-DM to ascertain if they could be novel cardiometabolic biomarkers for ACS-DM.

## Materials and Methods

### Ethics Approval of the Research Protocol

This study was approved by the ethics committee of the PLA General Hospital. All patients gave written informed consent.

### Participants

**FIGURE 1:**
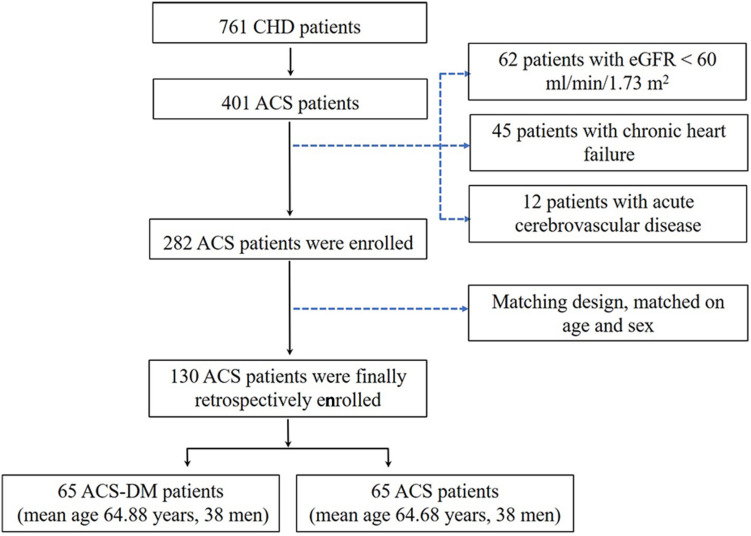
Study frame diagram. ACS, acute coronary syndrome; CHD, coronary heart disease; eGFR, estimated glomerular filtration rate.

A total of 761 patients with CHD were admitted to the Cardiology Department of PLA General Hospital from March to August 2018. The inclusion criteria were: (i) patients aged 18 years or older and diagnosed as having ACS according to the ACS diagnostic criteria and (ii) patients had a complete clinical data record. According to the inclusion criteria, 401 adult patients were enrolled in the study. The exclusion criteria were: chronic heart failure, acute cerebrovascular disease, moderate-to-severe renal dysfunction (estimated glomerular filtration rate (eGFR) < 60 mL/min/1.73 m^2^), prolonged bedridden status, mental illness, and malignant tumor. Considering the effect of disease on ceramide levels, 45 patients with chronic heart failure and 12 patients with acute cerebrovascular disease were excluded. After excluding another 62 patients with moderate-to-severe renal dysfunction, 282 participants with ACS were enrolled in the study. Among these, there were 65 patients with ACS-DM, who were selected as the experimental group (ACS-DM group), and 65 patients with only ACS (ACS group), who were selected as the control group after matching for age and sex with patients with ACS-DM. According to a previous study (Yao et al., 2019), ceramide levels were associated with ACS with an odds ratio (OR) of 3.0. According to the sample size calculation formula for a paired case–control study, the sample size required was calculated to be 56 patients by using PASS software (proportioning, two related proportions; tests for two correlated proportions, McNemar test and OR). Consequently, 65 patients in each group met the sample size requirements; therefore, 130 patients were finally selected as the study cohort. Among them, 65 patients (mean age 64.88 years, range 34–84 years; 38 men) were in the ACS-DM group, and 65 patients (mean age 64.68 years, range 34–86 years; 38 men) were in the ACS group (Figure 1).

### Data Collection

Patients’ demographic characteristics, lifestyle information, and medication use were obtained by review of medical records. The definition of “smoking” is having smoked more than 1 cigarette per day for over 1 year. Patients’ body mass index (BMI) was also calculated, and blood pressure was measured using the right arm of seated participants by mercury sphygmomanometer.

### Variable Biomarkers Determination

Blood samples were obtained with EDTA anticoagulation between 6:00 and 7:00 after patients fasted overnight. Samples were stored at 4°C for less than 1 h, and then the plasma samples were frozen at –80°C. Concentrations of fasting blood glucose (FBG), uric acid, blood lipid, and C-reactive protein (CRP) were measured by enzymatic assays (Roche Diagnostics, Mannheim, Germany). The glycated hemoglobin A_1_c (HbA_1_c) level was tested using high-performance liquid chromatography. The concentration of creatinine was determined by an enzymatic assay (Roche Diagnostics) on an autoanalyzer (7600; Hitachi, Tokyo, Japan). N-terminal pro-type-B natriuretic peptide (NT-proBNP) was measured by an electro-chemiluminescence immunoassay (Roche Diagnostics). The concentration of high-sensitivity cardiac troponin T (hs-cTnT) was measured on an E170 autoanalyzer (Modular Analytics; Roche Diagnostics) (Xiao et al., 2017).

### Quantification of Ceramides

The levels of ceramides were determined from the available plasma samples (*n* = 130). We used liquid chromatography–tandem mass spectrometry (LC-MS/MS) to quantitatively and simultaneously measure the four circulating ceramides in human plasma (Kauhanen et al., 2016). In short, the deuterated internal standards were added to 10 μL plasma: D7-Cer16:0, D7-Cer18:0, D7-Cer24:0, and D7-Cer24:1. The plasma levels of Cer16:0, Cer18:0, Cer24:0, and Cer24:1 were quantified on a Triple Quad^TM^ 3200 (Sciex, Framingham, MA, United States). A 5 μL sample was added, then the ceramide was separated on an Acquity UPLC I-Class column (Waters, Milford, MA, United States) using a 5 min gradient. Quantification was evaluated by calibration line samples containing known amounts of synthetic Cer16:0, Cer18:0, Cer24:0, and Cer24:1 and the deuterated standards. We calculated and plotted the peak area ratios of ceramides to the corresponding deuterated form according to the added ceramide concentration. According to the measured peak area ratios, the plasma concentration of ceramides was calculated by the individual fitting linear regression equation. The final concentration of ceramide is expressed in μmol/L.

### Definition of Variables

ACS refers to unstable angina (UA), non-ST elevated myocardial infarction (NSTEMI), and ST elevated myocardial infarction (STEMI). The diagnostic criteria for ACS are according to a comprehensive examination of clinical characteristics, including biomarkers of myocardial injury and an electrocardiogram (ECG). Unstable angina is defined as ischemic symptoms without increased biomarkers but with transient changes on the ECG. Myocardial infarction (MI) refers to myocardial necrosis during acute myocardial ischemia. The difference between STEMI and NSTEMI is whether the ST segment is elevated or not on the ECG (Anderson et al., 2007; Levine et al., 2016).

Hypertension was defined as systolic blood pressure (SBP) ≥ 140 mmHg, diastolic blood pressure (DBP) ≥ 90 mmHg, or taking antihypertensive medication. The diagnostic criteria of DM were FBG ≥ 7.0 mmol/L, HbA_1_c ≥ 6.5%, non-fasting glucose ≥ 11.1 mmol/L, or taking antihyperglycemic medication (Cosentino et al., 2020). The eGFR was calculated using eGFR (mL/min/1.73 m^2^) = 175 × plasma creatinine^–1^.^234^ × age^–0^.^179^ × 0.79 (if female) (Ma et al., 2006).

### Statistical Analyses

Continuous variables were expressed as the mean ± standard deviation or median, and categorical variables were expressed as percentages. Differences in risk factors at baseline and clinical characteristics between patients with ACS and those with ACS-DM were compared by Chi-squared test or Student’s *t*-test. Continuous variables in the skewness distribution were compared with the Wilcoxon rank sum test. Pearson correlation coefficients between individual ceramides and traditional cardiovascular risk factors for the whole study population were calculated. Multiple logistic regression analysis was used to assess the relationship between the concentration of ceramide and the risk of ACS-DM. The ceramide concentration was logarithm transformed. The basic model was adjusted for age and sex. The multi-model was further adjusted for smoking, BMI, hypertension, SBP, uric acid, triglycerides (TG), high-density lipoprotein cholesterol (HDL-C), LDL-C, CRP, HbA_1_c, and lipid-lowering medication. The multi-model + hs-cTnT was further adjusted for hs-cTnT based on the multi-model. The receiver operating characteristic (ROC) curve was used to assess the accuracy of the ceramide concentration for the prediction of ACS-DM; the discrimination was assessed with the area under curve (AUC).

Analyses were implemented with SPSS 23.0 (IBM, NY, United States). *P* < 0.05 was regarded as significant.

## Results

### Clinical Characteristics at Baseline

**FIGURE 2:**
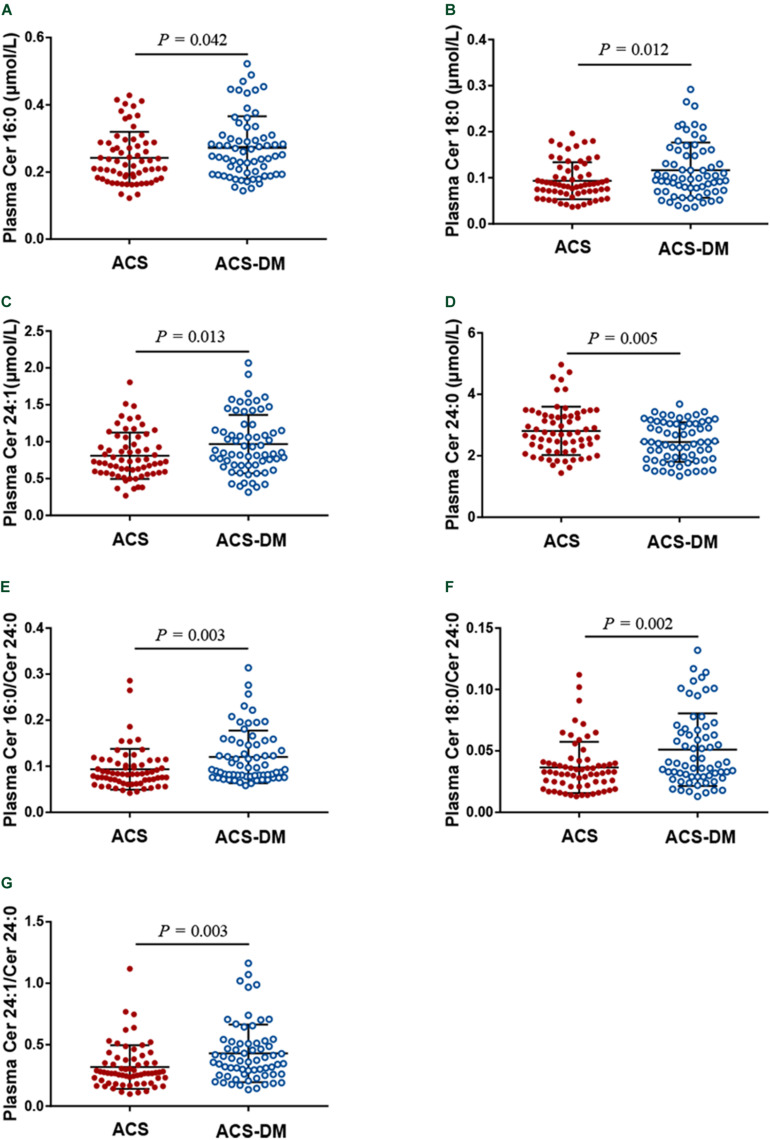
**(A–G)** A comparison of the levels of ceramides in patients with acute coronary syndrome (ACS) and those with comorbid acute coronary syndrome and type 2 diabetes mellitus (ACS-DM).

A total of 130 patients were enrolled in this study. The characteristics of the patients according to disease status (ACS group and ACS-DM group) are summarized in Table 1. Higher plasma levels of glucose, uric acid, TG, and CRP and lower levels of DBP and HDL-C were associated with ACS-DM. The proportion of patients with MI between the ACS group and the ACS-DM group did not differ, but the levels of hs-cTnT were higher in the ACS-DM group. Compared with the ACS group, the levels of Cer16:0, Cer18:0, and Cer24:1 and their ratios to Cer24:0 in the ACS-DM group were higher, whereas the level of Cer24:0 was lower (Figure 2).

**TABLE 1:** Clinical characteristics of the subjects categorized by disease status.

**Characteristic**	**ACS group (*n* = 65)**	**ACS-DM group (*n* = 65)**	***P*-value**
**Demographic**			
Age (years)	64.68 ± 10.17	64.88 ± 10.71	0.913
Male, *n* (%)	38 (58.5)	38 (58.5)	1.000
BMI (kg/m^2^)	25.32 ± 3.80	25.16 ± 3.70	0.817
Smoking, *n* (%)	33(50.8)	34(52.3)	0.861
MI, *n* (%)	32(49.2)	33(50.8)	0.861
Stroke, *n* (%)	8(12.3)	12(18.5)	0.331
Family history of CHD, *n* (%)	8(12.3)	15(23.1)	0.108
Hypertension, *n* (%)	41(63.1)	49(75.4)	0.128
Lipid-lowering medication, *n* (%)	49 (75.4)	51 (78.5)	0.677
Antihypertensive medication, *n* (%)	44 (67.7)	48 (73.8)	0.441
Systolic BP (mmHg)	133.0 ± 21.2	131.9 ± 19.2	0.755
Diastolic BP (mmHg)	76.5 ± 12.2	71.5 ± 10.7	0.014
**Laboratory value**			
TC (mmol/L)	3.80 ± 1.04	3.94 ± 1.22	0.484
TG (mmol/L)	1.22 ± 0.56	1.78 ± 1.07	< 0.001
HDL-C (mmol/L)	1.14 ± 0.27	1.00 ± 0.29	0.005
LDL-C (mmol/L)	2.37 ± 1.01	2.45 ± 1.06	0.652
FBG (mmol/L)	5.49 ± 0.70	10.75 ± 4.68	< 0.001
HbA_1_c (%)	5.74 ± 0.42	7.70 ± 1.56	< 0.001
Uric acid (μmol/L)	324.11 ± 97.56	363.61 ± 116.06	0.038
CRP (mg/dL)	0.81 (0.01, 9.28)	1.64 (0.01, 16.00)	0.023
hs-cTnT (ng/mL)	1.494 (0.003, 31.490)	3.223 (0.005, 57.240)	0.019
NT-proBNP (pg/mL)	2490.2 (35.8, 35 000.0)	2833.4 (21.3, 35 000.0)	0.766
eGFR (mL/min/1.73 m^2^)	95.3 ± 28.6	100.2 ± 76.3	0.739

*Values are reported as *n* (%), mean ± SD, or median (interquartile range). BMI, body mass index; MI, myocardial infarction; CHD, coronary heart disease; BP, blood pressure; TC, total cholesterol; TG, triglyceride; HDL-C, high-density lipoprotein cholesterol; LDL-C, low-density lipoprotein cholesterol; FBG, fasting blood glucose; HbA_1_c, glycated hemoglobin A_1_c; CRP, C-reactive protein; hs-cTnT, high-sensitivity cardiac troponin T; NT-proBNP, N-terminal pro-type-B natriuretic peptide; eGFR, estimated glomerular filtration rate.*

### Subgroup Analyses

**FIGURE 3:**
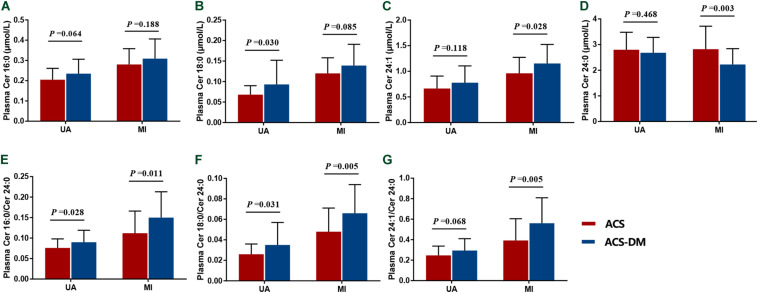
**(A–G)** Subgroup analysis of the levels of ceramides in patients with acute coronary syndrome (ACS) compared with those with comorbid acute coronary syndrome and type 2 diabetes mellitus (ACS-DM). MI, myocardial infarction; UA, unstable angina.

According to whether or not they had MI, patients were divided into the UA and MI subgroups and circulating ceramides in patients with ACS and those with ACS-DM were compared. In the UA subgroup, the levels of Cer18:0, Cer24:1, and Cer16:0/Cer24:0 in patients with ACS-DM were higher (*n* = 32) than those in patients with ACS (*n* = 33) (*P* < 0.05). In the MI subgroup, compared with patients with ACS (*n* = 32), the levels of Cer24:1, Cer16:0/Cer24:0, Cer18:0/Cer24:0, and Cer24:1/Cer24:0 were higher in patients with ACS-DM (*n* = 33), and the levels of Cer24:0 were lower in patients with ACS-DM (*P* < 0.05) (Figure 3).

### Correlation of Ceramides and Traditional Cardiovascular Risk Factors

The Pearson correlation coefficients for the levels of individual ceramides and those for traditional cardiovascular risk factors for the whole study population were calculated. Correlation analysis demonstrated that the levels of ceramides and their ratios had a mild-to-moderate correlation with those of traditional cardiovascular risk factors. Of note was the relatively strong correlation of Cer18:0 and Cer24:1 with the levels of CRP, blood lipids, FBG, HbA_1_c, and hs-cTnT (Table 2).

**TABLE 2:** Spearman rank correlation coefficients between ceramides and traditional cardiovascular risk factors for the whole study population (*n* = 130).

	**Age**	**TC**	**TG**	**HDL-C**	**LDL-C**	**CRP**	**FBG**	**HbA_1_c**	**NT-proBNP**	**hs-cTnT**
Cer(d18:1/16:0)	n.s.	0.401**	0.280**	–0.176	0.432**	0.349**	0.315**	0.235**	0.438**	0.440**
Cer(d18:1/18:0)	n.s.	0.340**	0.285**	−0.212*	0.395**	0.361**	0.306**	0.255**	0.390**	0.496**
Cer(d18:1/24:1)	n.s.	0.317**	0.280**	−0.277**	0.357**	0.370**	0.363**	0.300**	0.415**	0.414**
Cer(d18:1/24:0)	−0.307**	0.266**	0.203*	n.s.	0.276**	−0.260**	−0.200*	n.s.	−0.286**	−0.186*
Cer(d18:1/16:0)/Cer(d18:1/24:0)	0.224*	n.s.	n.s.	−0.217*	n.s.	0.431**	0.370**	0.240**	0.556**	0.455**
Cer(d18:1/18:0)/Cer(d18:1/24:0)	n.s.	n.s.	n.s.	−0.245**	0.204*	0.417**	0.341**	0.246**	0.475**	0.494**
Cer(d18:1/24:1)/Cer(d18:1/24:0)	n.s.	n.s.	n.s.	−0.273**	n.s.	0.420**	0.388**	0.289**	0.498**	0.429**

*P-value for comparison <0.05, **P-value for comparison <0.01. Non-significant correlation (*P* > 0.05) is marked with n.s. TC, total cholesterol; TG, triglyceride; HDL-C, high-density lipoprotein cholesterol; LDL-C, low-density lipoprotein cholesterol; CRP, C-reactive protein; FBG, fasting blood glucose; HbA_1_c, glycated hemoglobin A_1_c; NT-proBNP, N-terminal pro-type-B natriuretic peptide; hs-cTnT, high-sensitivity cardiac troponin T.

### Relationship Between Ceramides and the ACS-DM Group

Stepwise multiple logistic regression analysis revealed that the ceramide levels are significantly related to ACS-DM. In the basic model and the multi-model, the relationship between the ceramide level and the ACS-DM group was significant (*P* < 0.05). In the multi-model + hs-cTnT, Cer(d18:1/18:0) [OR 2.396; 95% confidence interval (CI) 1.103–5.205; *P* = 0.027], Cer(d18:1/24:1) (OR 2.826; 95% CI 1.158–6.896; *P* = 0.023), Cer(d18:1/18:0)/Cer(d18:1/24:0) (OR 2.242; 95% CI 1.103–4.555; *P* = 0.026), and Cer(d18:1/24:1)/Cer(d18:1/24:0) (OR 2.673; 95% CI 1.225–5.836; *P* = 0.014) were positively correlated with ACS-DM and Cer(d18:1/24:0) (OR 0.200; 95% CI 0.051–0.778; *P* = 0.020) was negatively correlated with ACS-DM, whereas the association of Cer16:0 and Cer16:0/Cer24:0 with ACS-DM disappeared. The significance of the ceramide levels in the adjusted models (basic model, multi-model, and multi-model + hs-cTnT) is presented in Table 3.

**TABLE 3:** Association between plasma ceramides and the risk of ACS-DM.

	**Model**	**OR**	**95% CI**	***P*-value**
Cer(d18:1/16:0)	Basic	3.214	1.038–9.955	0.043
	Multi	3.167	1.017–9.863	0.047
	Multi + hs-cTnT	0.397	0.051–3.120	0.380
Cer(d18:1/18:0)	Basic	2.270	1.063–4.847	0.034
	Multi	2.227	1.040–4.771	0.039
	Multi + hs-cTnT	2.396	1.103–5.205	0.027
Cer(d18:1/24:1)	Basic	2.741	1.135–6.616	0.025
	Multi	2.698	1.115–6.530	0.028
	Multi + hs-cTnT	2.826	1.158–6.896	0.023
Cer(d18:1/24:0)	Basic	0.191	0.052–0.698	0.012
	Multi	0.187	0.050–0.708	0.014
	Multi + hs-cTnT	0.200	0.051–0.778	0.020
Cer(d18:1/16:0) /Cer(d18:1/24:0)	Basic	4.614	1.785–11.924	0.002
	Multi	4.084	1.486–11.219	0.006
	Multi + hs-cTnT	1.531	0.295–7.940	0.612
Cer(d18:1/18:0) /Cer(d18:1/24:0)	Basic	2.825	1.438–5.550	0.003
	Multi	2.425	1.153–5.101	0.020
	Multi + hs-cTnT	2.242	1.103–4.555	0.026
Cer(d18:1/24:1) /Cer(d18:1/24:0)	Basic	3.263	1.544–6.897	0.002
	Multi	2.824	1.307–6.100	0.008
	Multi + hs-cTnT	2.673	1.225–5.836	0.014

*The table shows the OR and 95% CI of the incidence of comorbid acute coronary syndrome and type 2 diabetes mellitus (ACS-DM) associated with an increase in plasma log-transformed ceramides. The ORs were estimated from analyses using logistic regression. The basic model was adjusted for age and sex. The multi-model was futher adjusted for hypertension, smoking, BMI, systolic BP, uric acid, TG, HDL-C, LDL-C, CRP, HbA1c, lipid-lowering medication. OR, odds ratio; CI, confidence interval; Cer, Ceramide; hs-cTnT, highly sensitive cardiac troponin T; BMI, body mass index; BP, blood pressure; TG, triglycerides; HDL-C, high-density lipoprotein cholesterol; LDL-C, low-density lipoprotein cholesterol; CRP, C-reactive protein; HbA_1_c, glycated hemoglobin A_1_c.*

### Analyses of ROC Curves on Ceramide Levels in Patients With ACS-DM

**FIGURE 4:**
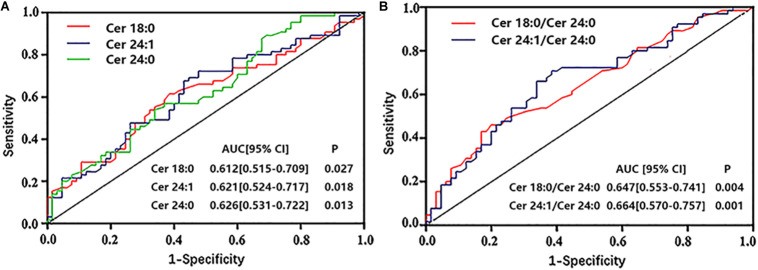
**(A,B)** Receiver operator characteristic (ROC) curves of ceramides to predict comorbid acute coronary syndrome and type 2 diabetes mellitus. ROC analysis was performed to determine the sensitivity and specificity of the value. AUC, area under the curve; CI, confidence interval.

Analyses of ROC curves indicated that the level of ceramides (except for Cer16:0 and Cer16:0/Cer24:0) had reasonable accuracy for the prediction of ACS-DM (Figures 4A,B).

## Discussion

We demonstrated in patients with ACS-DM and patients with ACS matched by age and sex that ceramides were associated with patients with ACS-DM independent of sex, age, or other risk factors of vascular disease. The levels of Cer18:0 and Cer24:1 and the ratios to Cer24:0 had reasonable accuracy for ACS-DM prediction. Cer18:0 and Cer24:1 and their ratios to Cer24:0 were positively correlated with ACS-DM, and Cer24:0 was negatively correlated with ACS-DM. These results suggest that ceramides could be new biomarkers of ACS-DM.

Recent studies have demonstrated that ceramides are associated with an atherosclerotic process (Bismuth et al., 2008). Elevated levels of ceramides are also related to multiple cardiovascular risk factors, such as hypertension (Mielke et al., 2019), heart failure (Lemaitre et al., 2019), and DM (Neeland et al., 2018). Increases in levels of ceramides are predictive of cardiovascular events and are also independently correlated with adverse cardiovascular events in patients with and without CHD. The predictive value of ceramides is better than that of LDL-C levels (Meeusen et al., 2018). Gierens et al. explained the potential cause of this phenomenon. They confirmed that interleukin-6 (IL-6) enhances LDL receptor activity in the liver during the inflammatory response, subsequently increasing the elimination of circulating LDL-C. Thus, the acute inflammatory response in patients with ACS may increase the clearance of LDL-C, leading to a decrease in the circulating LDL-C concentration and affecting the accuracy of risk prediction (Lubrano et al., 2015). Determination of ceramide levels using a high-throughput methodology such as LC-MS/MS is efficient and convenient. Accurate quantification of ceramides can be achieved by isotope-labeled standards, enabling ceramides to be new potential predictors of cardiovascular events. Long-chain ceramides have the effect of promoting apoptosis, and very-long-chain ceramides (Cer24;0) have an anti-apoptotic effect (Grösch et al., 2012). Consistent with the present study, long-chain species (Cer16:0 and Cer18:0) increased cardiovascular risk, and very-long-chain (Cer24:0) species were cardioprotective. Also, in patients with established CHD, Cer24:0 appears to be cardioprotective, and the increase in Cer24:0/Cer24:1 can lower the risk of cardiovascular events in patients with or without DM (Menuz et al., 2009). Our results are consistent with these studies.

Our study demonstrated that the levels of Cer16:0, Cer18:0, and Cer24:1 and their ratios to Cer24:0 in patients with ACS-DM were higher than those in patients with ACS. The levels of Cer24:0, Cer18:0/Cer24:0, and Cer24:1/Cer24:0 had a relatively strong correlation with ACS-DM. The levels of Cer18:0 and Cer24:1 were associated with the levels of CRP, blood lipids, FBG, and HbA_1_c; these results are consistent with a previous study (Havulinna et al., 2016). Cer18:0 and Cer24:1 may be the main risk factors and pathogenic factors for ACS-DM. The Cer24:0 level was negatively correlated with ACS-DM and appeared to have a protective effect in patients with ACS-DM.

Ceramides are complex sphingolipids widely expressed in cells and lipoproteins. The increase in ceramide content in LDL promotes the atherosclerotic process of the vascular wall (Li et al., 2014). Ceramides are involved in biological processes that may influence atherosclerosis and cardiovascular events, including oxidative stress apoptosis, endothelial dysfunction, inflammation, lipotoxicity, and insulin resistance (Alessenko et al., 2018). The inflammatory cytokines interferon-γ and IL-6 promote ceramide synthesis. Ceramides participate in endothelial damage and platelet activation (Marathe et al., 1998). Ceramides are associated with vulnerable atherosclerotic plaques and participate in the process of atherosclerosis, including lipoprotein aggregation and uptake, the inflammatory response, endothelial damage, and apoptosis. They also inhibit the production of apolipoprotein E in macrophages and lead to cholesterol accumulation in macrophage-derived foam cells (Lucic et al., 2007). The levels of ceramides increased with accumulated lipid loading and inflammation in patients with DM, and ceramides can directly regulate β-cell apoptosis and induce insulin resistance (Kayser et al., 2019). Circulating leptin levels were significantly increased in insulin resistance, which is associated with cardiac dysfunction (Ren and Relling, 2006). The potential toxicity of ceramides and their ability to inhibit contraction in cardiomyocytes are related to exposure time (Relling et al., 2003). Several studies have demonstrated that different ceramide species have different effects. For instance, cell experiments have shown that Cer16:0 can promote apoptosis, while Cer24:0 appears to protect against apoptosis. Evidence is also accumulating on the functions of ceramides with different chain lengths. Increases in Cer16:0 but not Cer24:0 are involved in insulin resistance. C2 ceramide reduced the damage to cardiac function caused by a high glucose concentration (Colligan et al., 2002). In brief, ceramides have a mutual pathologic mechanism in patients with ACS and those with DM that increases the risk of cardiovascular events. Ceramides may be potential targets for the treatment of CHD in patients with DM.

## Limitations

There were four main limitations to this study. First, our study was an observational study, unable to establish a causal relationship between ceramides and ACS-DM. Because of this, it is unclear whether cardiovascular risk treatment based on ceramides would improve clinical benefits. Second, although a paired design was used to reduce confounding factors, the sample size was relatively small. The possibility of residual confounding from imprecisely measured or unmeasured variables cannot be ruled out. Third, our study cohort comprised primarily a Chinese Han population. Therefore, it is necessary to conduct further research on an ethnically more diverse population. Fourth, the AUC showed that the individual discriminative power of ceramides was low for ACS-DM detection (AUC < 0.70). Therefore, our observations need to be further confirmed in longitudinal studies with a larger sample.

## Conclusion

In conclusion, circulating ceramides are positively correlated with the risk of ACS-DM comorbidity. However, longitudinal follow-up studies are still needed to evaluate the causal relationship between ceramides and comorbid ACS-DM. The results of this study demonstrated new insights into the pathogenesis of comorbid ACS-DM. Circulating ceramides may provide new options for risk evaluation as novel biomarkers of cardiometabolic diseases.

## Data Availability Statement

The raw data supporting the conclusions of this article will be made available by the authors, without undue reservation.

## Ethics Statement

The studies involving human participants were reviewed and approved by The ethics committee of Chinese PLA General Hospital (Beijing, China). The patients/participants provided their written informed consent to participate in this study.

## Author Contributions

FC designed the experiments. RC, ZF, SL, MX, JZ, DH, WH, and LY performed the experiments. RC, ZF, and LY analyzed the data. RC prepared the manuscript. RC, YW, and LF supervised data collection. All authors have contributed to the study and approved the manuscript.

## Conflict of Interest

The authors declare that the research was conducted in the absence of any commercial or financial relationships that could be construed as a potential conflict of interest.
